# Analysis of cellular senescence-related genes in calcified aortic valve disease and the potential therapeutic role of β-Carotene

**DOI:** 10.1371/journal.pone.0318574

**Published:** 2025-03-10

**Authors:** Yijing Tao, Chengjie Gao, Juan Wang, Qiyin Zhang, Zhisong Wang, Leng Han, Donglai Cao, Qianwen Yao

**Affiliations:** 1 Department of Cardiology, Changshu Hospital Affiliated to Soochow University, Changshu No.1 People’s Hospital, Changshu, China,; 2 Department of Geriatrics, Shanghai Sixth People’s Hospital Affiliated to Shanghai Jiao Tong University School of Medicine, Shanghai, China,; 3 Department of Cardiology, The Fourth Affiliated Hospital of Soochow University, Suzhou Dushu Lake Hospital, Medical Center of Soochow University, Suzhou, China; Nanjing First Hospital, Nanjing Medical University, CHINA

## Abstract

**Objective:**

Calcific aortic valve disease (CAVD) is a progressive, age-related degenerative disease characterized by the accumulation of calcium deposits in the aortic valve. We aim to screen key genes associated with cellular senescence (CS) in CAVD.

**Methods:**

The GSE12644 and GSE51472 datasets from the GEO database was utilized in this study, and differentially expressed genes (DEGs) were identified using the “*limma*” R package. CS-related DEGs (CS-DEGs) were determined through the CellAge database. Gene Ontology (GO) and Kyoto Encyclopedia of Genes and Genomes (KEGG) enrichment analyses were performed on CS-DEGs. A protein–protein interaction (PPI) network was constructed using the STRING database. The cytoHubba plug-in in Cytoscape was used to identify hub genes. A noncoding-RNA-mRNA regulatory network was established. DSigDB database was used to to identify drugs potentially be useful for treating CAVD.

**Results:**

A total of 16 CS-DEGs were identified. These genes were primarily associated with collagen metabolic process, collagen catabolic process and external side of plasma membrane. 10 hub genes were identified as regulators of cellular senescence in CAVD: LPAR1, PTPN6, CD28, ID1, MEIS2, FGFR3, KDR, MMP7, AR, HIF1A. The Noncoding RNA-mRNA regulatory network indicated that CS-DEGs may be regulated by noncoding RNAs. β-Carotene, a naturally occurring carotenoid with antioxidant properties, was identified potential therapeutic agents through interacting with MMP9, MEIS2, and CTSB.

**Conclusion:**

This study provides insights into the key genes and pathways related to cellular senescence in CAVD (MMP9, MEIS2, and CTSB) and highlights the potential role of β-Carotene treatment of CAVD.

## Introduction

Cellular senescence refers to the process by which cells gradually lose their ability to divide and proliferate, with core mechanisms typically thought to include telomere shortening and DNA damage [1,2]. This process not only accompanies a progressive decline in cellular function but also has profound implications for the overall health of the organism [3,4]. Cellular senescence is a critical factor in a variety of age-related diseases, such as Alzheimer’s disease, cardiovascular diseases, and osteoporosis [5,6]. As the global population continues to age, the question of how to delay cellular senescence and improve the quality of life for the elderly has become a major focus of medical research, with significant implications for the sustainable use of healthcare resources [7,8].

In the cardiovascular system, the accumulation of senescent cells plays a pivotal role in the progression of cardiovascular diseases, particularly those associated with aging, such as coronary artery disease, vascular calcification, and valvular calcification [9–11]. Senescent cells not only obstruct normal cellular renewal through intrinsic mechanisms like telomere shortening but also secrete large quantities of inflammatory factors, extracellular matrix proteins, and enzymes, which trigger localized inflammatory responses and tissue damage [12–14]. This localized inflammation and tissue damage further compromise the function and structure of the cardiovascular system, accelerating the progression of cardiovascular diseases [15,16]. Therefore, in-depth research into the mechanisms of cellular senescence and the exploration of effective interventions are essential for slowing the progression of cardiovascular diseases and improving the overall health of the elderly population. Beta-carotene is a natural antioxidant, commonly found in various fruits and vegetables. As an antioxidant, beta-carotene helps neutralize free radicals, thereby reducing oxidative stress, which is a key contributor to many degenerative diseases. Its effects on cellular senescence and apoptosis may offer new avenues for managing CAVD [17].

In the present study, we conduct a bioinformatic analysis based on GEO database to identify important cellular senescence related genes in the occurrence and development of calcified aortic valve disease (CAVD). CAVD is an aging-related disease and has a high prevalence among the elderly, the senescence related genes in this analysis may be potential therapy targets for preventing and treatment of CAVD. We also found that β-Carotene may have favor effects through interacting with senescence related genes.

## Methods

### GEO datasets collection

Two CAVD related datasets, including GSE12644 and GSE51472, were obtained from Gene Expression Omnibus (GEO) database (https://www.ncbi.nlm.nih.gov/geo/; Lastly accessed data: 2024-8-31). GEO database is a publicly accessible repository that archives a multitude of high-throughput sequencing and microarray datasets contributed by research institutions worldwide [18–20]. Datasets included in this study were both generated using the GPL570 platform.

### Data preprocessing

The “*limma*” R package was employed to conduct normalization and the “*sva*” R package was employed to conduct integration of GSE12644 and GSE51472 and batch effect correction. The “*limma*” R package was also used to analyze the differential expression of mRNAs. Differential expression was determined using thresholds of adjusted *p* <  0.05 and | logFC | ≥  0.5. Probe sets lacking corresponding gene symbols were excluded, and genes associated with multiple probe sets were averaged.

### Identification of cellular senescence (CS) related genes

A total of 866 genes related to CS were obtained from the CellAge database (https://genomics.senescence.info/cells/), a resource curated from genetic manipulation experiments conducted across various human cell types. The CellAge database compiles both experimental evidence and literature-based data to annotate genes involved in cellular senescence. Specifically, the database integrates findings from transcriptomic, proteomic, and functional assays. The overlap between these CS-related genes from the CellAge database and the differentially expressed genes (DEGs) identified in GSE12644 and GSE51472 was used to define the CS-related DEGs.

### GO and KEGG enrichment analysis

“clusterProfiler” R package was adopted to conducted Gene Ontology (GO) and KEGG pathway analysis to further understand the primary biological functions of CS-DEGs. Adjusted p-value < 0.05 was set as significant threshold.

### Gene Set Enrichment Analysis (GSEA)

To enhance our comprehension of the biological processes the CS-DEGs enriched in, we performed Gene Set Enrichment Analysis (GSEA) utilizing the clusterProfiler” R package. Gene sets corresponding to Hallmark and Canonical Pathways were sourced from the MSigDB collections. Statistical significance was determined using an adjusted p-value threshold of < 0.05 and a false discovery rate (FDR, or q-value) threshold of < 0.25.

### PPI network construction

The STRING website was used to investigate the interactions among proteins, including direct binding relationships and co-regulatory pathways, to construct a protein–protein interaction (PPI) network characterized by intricate regulatory connections. Interactions with a combined score exceeding 0.4 were considered statistically significant. The visualization of this PPI network was performed using Cytoscape. To identify key functional modules within the network, the Molecular Complex Detection (MCODE) plug-in for Cytoscape was employed with the following selection criteria: K-core =  2, degree cut-off =  2, maximum depth =  100, and node score cut-off =  0.2.

### Identification of hub CS-DEGs

CS-DEGs were identified using the “cytoHubba” plug-in in Cytoscape. We employed six widely recognized algorithms—MCC (Maximum Clique Centrality), MNC (Maximum Neighborhood Component), Degree, Closeness, Radiality, and EPC (Edge Percolated Component)—to evaluate and prioritize these core CS-DEGs. Following this, a co-expression network of the identified hub genes was constructed using GeneMANIA, a reliable tool for detecting internal relationships within gene sets.

### Noncoding RNA-mRNA regulatory network

miRNA-target interactions were retrieved from miRWalk, a publicly accessible database specializing in miRNA-target interactions. Upstream lncRNAs were predicted based on TargetScan, miRDB and miRTarBase database. Finally, Noncoding RNA-mRNA regulatory network were integrated using Cytoscape.

### Identification of Potential therapeutic agents for CAVD

The DSigDB is an extensive repository of drug-gene interactions and drug-effect signatures. We utilized the online platform Enrichr to access the DSigDB, which enabled us to identify drugs potentially be useful for treating CAVD. The interaction of β-Carotene with was predicted by CB-DOCK2 website.

## Results

### Identification of differentially expressed genes (DEGs)

**Fig 1:**
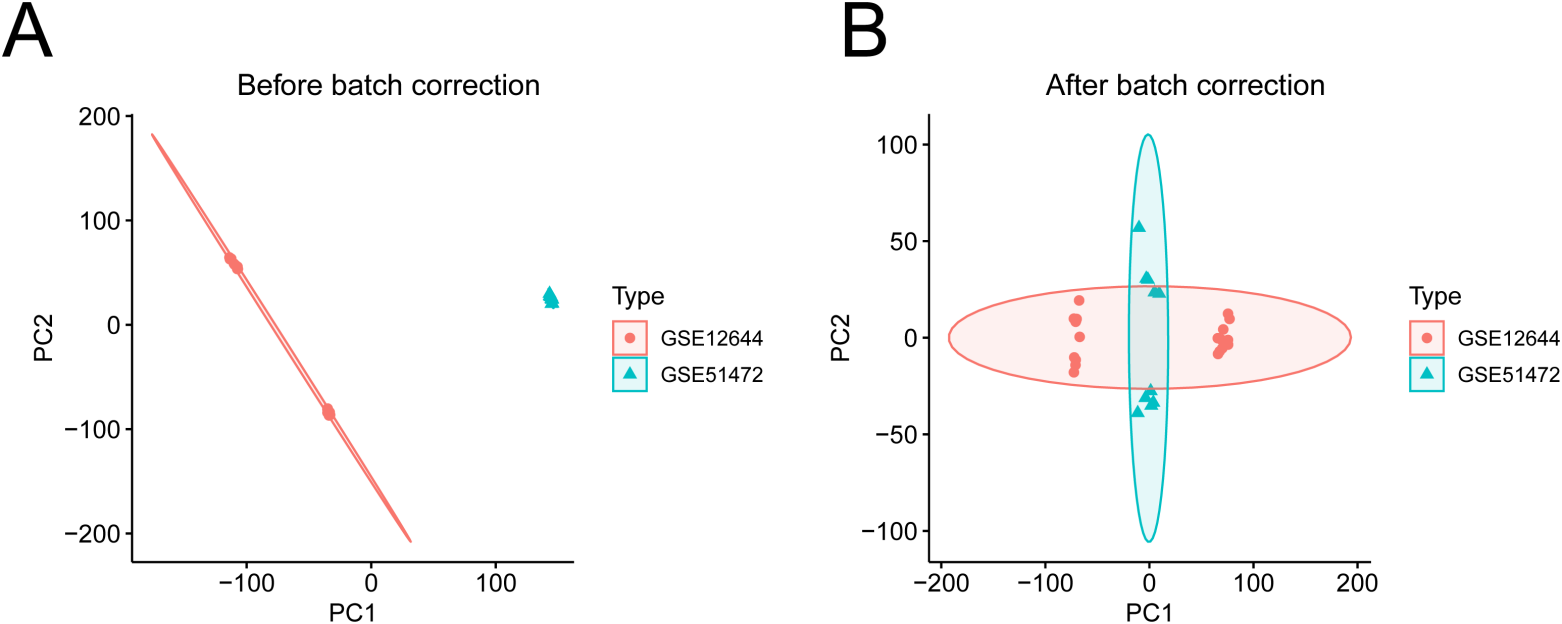
PCA plot before (A) and after (B) batch effects correction.

**Fig 2:**
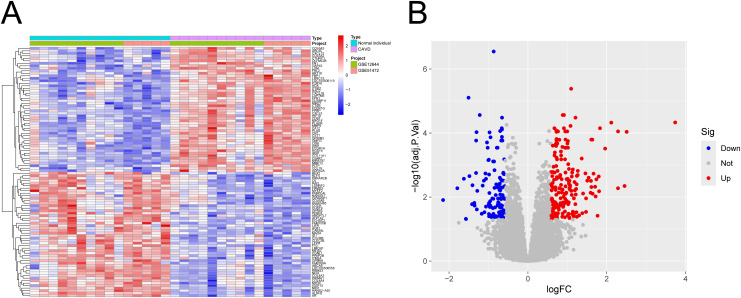
Identification of DEGs in CAVD. (A) Heatmap visualizing the DEGs in CAVD; (B) Volcano plot visualizing the DEGs in CAVD.

**Fig 3:**
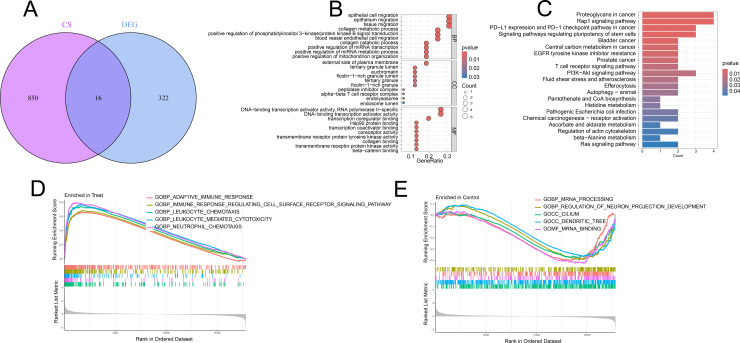
Identification of CS-DEGs and functional enrichment analysis. (A) Venn plot intersecting CS-related genes from CellAge database and DEGs in CAVD; (B) GO enrichment analysis of CS-DEGs; (C) KEGG enrichment analysis of CS-DEGs; (D-E) GSEA enrichment analysis of CS-DEGs.

Fig 1 illustrates the research workflow for this study. Considering the different experimental conditions of the datasets included in the present study (GSE12644 and GSE51472), we firstly conducted batch effect correction, and the successful correction of batch effects was visualized by PCA plot (Fig 2A-B). Following the normalization and batch effect correction, a total of 338 DEGs were identified (Fig 3A). Of them, 123 DEGs were up-regulated and 215 DEGs were down-regulated (Fig 3B).

### Identification of cellular senescence related DEGs (CS-DEGs)

**Fig 4:**
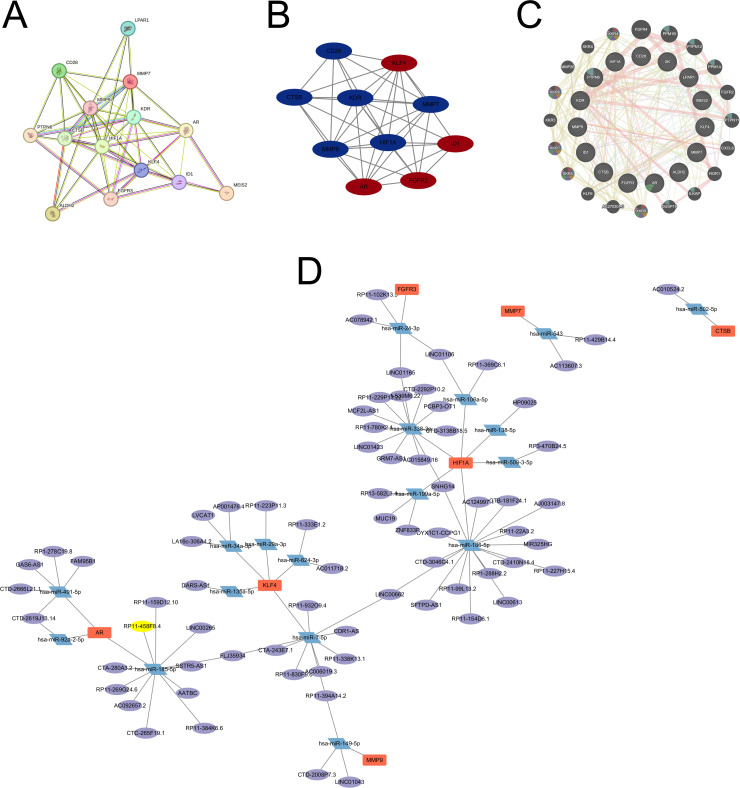
PPI network and noncoding RNA-mRNA regulatory network construction. (A) PPI network by string website; (B) a tightly interconnected gene modules identified by MCODE plug-in; (C) PPI network by GeneMANIA database; (D) noncoding RNA-mRNA regulatory network.

Venn diagram analysis revealed 16 CS-DEGs that were common to both the merge datasets of GSE12644 and GSE51472 and the CellAge database (Fig 4A). These CS-DEGs were ALDH2, AR, MMP9, MEIS2, CTSB, HIF1A, KLF4, ID1, CD28, CKAP2, CTNNAL1, LPAR1, FGFR3, PTPN6, MMP7, and KDR.

### Functional enrichment analysis

To further identify the biological functions of the CS-DEGs in CAVD, we carried out Gene Ontology (GO) Functional enrichment analysis, KEGG pathways enrichment analysis, and Gene Set Enrichment Analysis (GSEA) based on the 16 CS-DEGs in CAVD in the present study. The GO analysis (Fig 4B) revealed that CS-DEGs were prominently enriched in biological processes related to collagen metabolic process, collagen catabolic process, and positive regulation of phosphatidylinositol 3 − kinase/protein kinase B signal transduction. In terms of cellular components, these DEGs were predominantly associated with tertiary granule lumen, euchromatin, and external side of plasma membrane. For molecular functions, the DEGs were chiefly linked to transcription coregulator binding, DNA−binding transcription activator activity, RNA polymerase II−specific, and Hsp90 protein binding. Additionally, KEGG pathway analysis (Fig 4C) indicated that the DEGs were significantly involved in pathways related to proteoglycans in cancer, Rap1 signaling pathway, and the PD − L1 expression and PD − 1 checkpoint pathway in cancer signaling pathway. The GSEA identified the top five significantly enriched categories as adaptive immune response, immune response-regulating cell surface receptor signaling, leukocyte chemotaxis, leukocyte-mediated cytotoxicity, and neutrophil chemotaxis (Fig 4D-E).

### PPI network construction and module analysis

**Fig 5:**
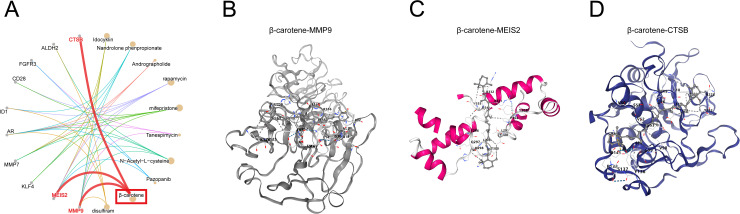
Potential therapeutic **β-carotene for CAVD**. (A) Potential therapeutic agents targeting CS-DEGs for CAVD analyzed by DSigDB database; (B-D) β-Carotene interacting with MMP9, MEIS2, and CTSB predicted by CB-DOCK2 website.

The protein-protein interaction (PPI) network for CS-DEGs with combined scores greater than 0.4 was constructed using Cytoscape, resulting in a network comprising 14 nodes and 56 interaction pairs (Fig 5A). The MCODE plug-in for Cytoscape also identified a tightly interconnected gene modules, which included 10 CS-DEGs and 36 interaction pairs (Fig 5B). Employing the cytoHubba plug-in, we ultimately identified the top 10 hub genes, including LPAR1, PTPN6, CD28, ID1, MEIS2, FGFR3, KDR, MMP7, AR, HIF1A. Further analysis using the GeneMANIA database was conducted to explore the co-expression network and the functional relationships of these genes. The protein-protein interaction (PPI) network analysis revealed that 40.32% of the interactions were direct physical interactions, 16.68% were predicted interactions, 17.12% were attributed to co-localization, 8.98% were related to specific pathways, 11.2% were genetic interactions, and 5.7% were based on co-expression data (Fig 5C). These hub genes were found to play critical roles in regulating the plasma membrane phospholipid scrambling, phospholipid translocation, phagocytosis engulfment, apoptotic cell membrane, plasma membrane organization, regulation of membrane lipid distribution, phosphoprotein phosphatase activity.

### Noncoding RNA-mRNA regulatory network

We also constructed noncoding RNA-mRNA regulatory network for the CS-DEGs in CAVD. We found that 8 of the CS-DEGs in CAVD may be regulated by noncoding RNAs, including MMP9, HIF1A, KLF4, MMP7, AR, FGFR3, and CTSB. A total of 18 miRNAs and 60 lncRNAs were identified in the noncoding RNA-mRNA regulatory network, which will help of understanding underlying roles and mechanisms of CS-DEGs in CAVD (Fig 5D).

### Potential therapeutic agents targeting CS-DEGs for CAVD

In our analysis utilizing DSigDB, we identified 756 agents with potential therapeutic applications for CAVD (Fig 6A). Among these candidates, β-carotene was selected for in-depth examination. β-Carotene, a naturally occurring carotenoid with antioxidant properties, can neutralize free ROS, which damages cellular DNA, proteins, and lipids, ultimately accelerating cellular senescence. Our research indicates that β-Carotene’s modulation of cellular senescence through interacting with MMP9, MEIS2, and CTSB. These findings suggest that β-Carotene has considerable therapeutic potential for PE and should be further explored in clinical trials. Moreover, the interaction of β-Carotene with was predicted by CB-DOCK2 website, as shown in Fig 6B-D.

## Discussion

CAVD is a degenerative disorder whose incidence markedly increases with advancing age [21–23]. Epidemiological studies indicate that the prevalence of CAVD among the elderly numerous times greater than that observed in the general population [24]. The age-related rise in CAVD incidence is associated not only with hemodynamic changes and comorbid conditions but also with the senescence of various cell types within the aortic valve [25,26]. Research has demonstrated that cellular aging, including the senescence of aortic valve interstitial cells, endothelial cells, and fibroblasts, significantly contributes to the progression of CAVD. This study utilized datasets related to CAVD obtained from public GEO database (GSE12644 and GSE51472), to investigate the role of cellular senescence-related genes (CellAge database) in the development and progression of CAVD [27–29]. With the continuous advancement of sequencing technologies, an increasing number of researchers have deposited their sequencing data into public databases, greatly facilitating subsequent investigations into the mechanisms underlying various diseases. However, variations in measurement instruments and experimental conditions across different laboratories can lead to batch effects among datasets [30,31]. In our study, we first addressed and removed batch effects between the two datasets. We compared PCA plots before and after batch effect correction, demonstrating successful mitigation of batch effects. This improvement enables a more accurate investigation into the functions and roles of aging-related genes in CAVD.

Despite substantial advancements in pharmacological and interventional treatments over recent decades, issues related to medication side effects, surgical complications, and high costs persist, prompting ongoing research into alternative treatment strategies [32–34]. Numerous studies have investigated the therapeutic potential of natural compounds for CAVD. Recent basic research has highlighted that Morusin is a flavonoid and principal phenolic compound derived from the root bark of Morus alba, can mitigate aortic calcification by attenuating the senescence of aortic valve interstitial cells [35]. Cellular senescence is a fundamental biological process impacting human health and longevity, closely linked to the onset and progression of various chronic conditions, including neurodegenerative diseases, cardiovascular diseases, cancer, and diabetes [36]. Typically, cellular senescence involves cell cycle arrest mediated by the inhibition of cyclin-dependent kinases (CDKs) through the p53/p21 and/or p16 pathways in response to persistent DNA damage [37,38]. Senescent cells are characterized by four primary features: cell cycle arrest, a senescence-associated secretory phenotype (SASP), macromolecular damage, and metabolic dysfunction [39,40]. The SASP is a complex array of secreted cytokines, chemokines, growth factors, and proteases, with its composition varying significantly based on cell type, tissue environment, and senescence-inducing stimuli [41,42]. Cellular senescence significantly impacts bone metabolism and is implicated in degenerative bone and joint diseases [43]. Senescent cells disrupt the balance between bone formation and resorption through SASP factors, thereby contributing to osteoporosis and degenerative changes in bone tissue [44–46]. Research indicates that targeting the clearance of senescent cells or inhibiting their functions can effectively slow the progression of osteoporosis and degenerative joint diseases. In this study, we analyzed sequencing data from CAVD patients and the general population, identifying differentially expressed genes. By intersecting the differentially expressed genes associated with CAVD and those related to cellular senescence, we identified 16 genes that play a crucial role in cellular senescence and are categorized as cellular senescence-related differentially expressed genes (CS-DEGs). We also conducted functional enrichment analysis for these genes and, in addition, constructed a differential expression network and a potential regulatory network involving non-coding RNAs for these genes.

By mitigating oxidative stress, β-carotene protects cells from damage and potentially decelerates the cellular senescence process [47,48]. It also reduces apoptosis by minimizing oxidative stress and DNA damage. Additionally, β-carotene may inhibit inflammation by decreasing the production of pro-inflammatory factors, likely through downregulation of the NF-κB pathway, thereby reducing inflammatory mediator release [49,50]. Furthermore, β-carotene’s antioxidant properties can decrease mitochondrial oxidative stress, preserving mitochondrial function and integrity, which helps delay cellular aging associated with mitochondrial damage [51–53]. However, one key limitation of this study is the lack of biological validation, due to funding and resources limitations, biological validation could not be included in this study. However, we hope to carry out biological validation and explore detailed mechanisms in the future study. In this study, we also identified β-carotene as a notable natural compound with potential therapeutic effects on CAVD. It is posited that β-carotene may exert its effects by interacting with three molecules: MMP9, MEIS2, and CTSB, potentially modulating and slowing the progression of CAVD.

## Conclusion

In this study, we performed a comprehensive bioinformatic analysis to investigate the role of cellular senescence-related genes in calcified aortic valve disease (CAVD), an age-related condition prevalent among the elderly. By analyzing datasets from the GEO database and integrating data from the CellAge database, we identified 16 cellular senescence-related differentially expressed genes (CS-DEGs) that are significantly involved in CAVD. Functional enrichment analyses revealed that these genes are associated with various biological processes and pathways relevant to the disease, including collagen metabolism and immune response regulation.

## Ethics statement

This study utilized publicly available data from the Gene Expression Omnibus (GEO) database, which contains de-identified gene expression profiles. As the data used in this analysis are anonymized and publicly accessible, no ethical approval was required for the study. The authors confirm that all data analyzed were obtained in accordance with the ethical standards set by the respective data providers and were used following the guidelines for open-access datasets.
